# Veterinary Medical Association of New York County

**Published:** 1898-02

**Authors:** Robert W. Ellis

**Affiliations:** Secretary


Secretary.

### VETERINARY MEDICAL ASSOCIATION OF NEW YORK COUNTY.

THE regular monthly meeting of the Association was called to order Wednesday, January 5, 1898, at 8 P.M., with the President, Dr. Huidekoper, in the chair.

After roll-call, the minutes of the previous meeting were read and approved.

The Secretary then read the list of members appointed on the committees by the President, for the current year, as follows:

Board of Censors: H. D. Gill, chairman; J. S. Cattanach, Roscoe R. Bell, J. J. Foy, R. S. MacKellar.

Judiciary Committee: A. O'Shea, chairman; H. D. Gill, J. E. Ryder, C. C. Cattanach, Herbert Neher.

Reports of special committees: Dr. Gill, chairman of committee appointed by the chair to search the register, reported that Dr. Grenside was properly registered, and that the report in reference to Dr. Hingston as given at last meeting was correct. Moved and seconded, that the report be accepted; carried. The chair next reported in reference to the *Blue-book* as follows: It is practically all in type, except the table of contents, which will appear in the next ten days. President Huidekoper had advanced considerable money, and more would be required to be paid in the next ten days, so the following proposition was made by him to the Association: That if the Association so desired he would advance the amount required, when due, and that he would thereby assume the entire financial part of the *Blue-book*.

After some discussion it was moved and seconded that President Huidekoper have the financial control of the *Blue-book*; carried.

Reports of cases: Dr. Bell reported a case of fistula at the inferior border of the buccinator muscle. Free discussion followed. Dr. Gill spoke of his experience with azoturia cases after the Christmas holidays, having noted that all of his cases came from badly ventilated stables.

Moved by Dr. Hanson, that a committee of five be appointed to see what could be done to encourage veterinarians to attend and join the Veterinary Medical Association of New York County, and report at next meeting; seconded; carried.

Moved and seconded that the Executive Committee take into consideration the entertaining of the New York State Veterinary Medical Association; carried.

Moved and seconded that the meeting adjourn; carried.

ROBERT W. ELLIS, D.V.S.,
Secretary.

